# Acute Dysphagia Caused by Sarcomatoid Squamous Cell Carcinoma of the Esophagus

**DOI:** 10.7759/cureus.4129

**Published:** 2019-02-25

**Authors:** Benjamin J Phelps, Yale M Tiley, Jamie L Skrove, Andrew C Berry, Karthik Mohan

**Affiliations:** 1 Internal Medicine, Palmetto General Hospital, Hialeah, USA; 2 Internal Medicine, Larkin Community Hospital, South Miami, USA; 3 Gastroenterology, Larkin Community Hospital, South Miami, USA; 4 Gastroenterology, Palmetto General Hospital, Hialeah, USA

**Keywords:** spindle cell carcinoma, sarcomatoid, esophageal cancer, dysphagia, squamous cell cancer

## Abstract

Sarcomatoid squamous cell carcinoma of the esophagus is a rare etiology of esophageal cancer. Due to its large polypoid character, patients suffering from this disease typically present with progressive dysphagia, weight loss, odynophagia, or chest pain. Risk factors for esophageal cancer include smoking, alcohol use, and chronic gastroesophageal reflux disease. We present a case of an elderly female who presented to our hospital with a one-week history of progressive dysphagia secondary to a large esophageal sarcomatoid squamous cell carcinoma.

## Introduction

Esophageal cancer is a serious medical condition that carries a grave prognosis. It is the eighth most common cancer type and the sixth most common cause of cancer-related death with a five-year survival rate between 15%-30% [1]. The majority of esophageal cancers consist of squamous cell carcinomas and adenocarcinomas as these types account for greater than 90% of all diagnosed cases. In the United States, almost 18,000 new cases of esophageal cancer will be diagnosed each year. Risk factors including smoking and alcohol use predispose patients to this disease [1-2]. Chronic gastritis and esophageal reflux disease leading to Barrett’s esophagus, a metaplastic process in which squamous cells are replaced by simple columnar cells along the lining of the lower esophagus, also have the potential for malignant transformation. Sarcomatoid squamous cell carcinoma of the esophagus is a rare etiology of esophageal cancer, accounting for approximately 2% of all esophageal malignancies. [3] Histologically, esophageal sarcomatoid squamous cell carcinomas are biphasic; the epithelial component is usually limited to a few areas and the bulk of the tumor has a pleomorphic sarcomatoid appearance [3]. On gross examination, sarcomatoid squamous cell carcinoma presents as a bulky, pedunculated mass with a spindle cell component in the stroma and a squamous cell component on the surface [3]. It is suggested that although sarcomatoid squamous cell carcinoma carries a biphasic morphology, the disease stems from a single monoclonal origin.

Clinically, patients typically present with progressive dysphagia and weight loss, although symptoms may remain mild until the patient develops advanced disease. In a recent review, approximately 10% of cases are discovered in asymptomatic patients [4]. Gastrointestinal blood loss leading to iron deficiency anemia is also common. These cancers are diagnosed using endoscopy with biopsy, often requiring multiple biopsy sites to isolate pathologic tissue. Endoscopic ultrasound and positron emission tomography/computed tomography (PET/CT) imaging are the modalities of choice in staging the disease. Esophagectomy has historically been the treatment of choice for local, non-invasive esophageal tumors. For more advanced, inoperable disease, treatment is palliative, focused on the ability to restore swallowing and the ability to feed, often requiring gastrostomy tubes to sustain adequate nutrition [5]. In some cases, combination chemoradiation is used to slow tumor growth and prolong the need for palliative treatment. In this case report, we describe a 92-year-old female patient who presented with a one-week history of worsening dysphagia who was discovered to have a large esophageal sarcomatoid squamous cell carcinoma.

## Case presentation

A 92-year-old female patient with a past medical history of hypertension, hyperlipidemia, mechanical aortic valve replacement, coronary artery bypass to three vessels 15 years prior, and a history of breast cancer treated with mastectomy and radiation therapy presented with a one-week history of progressive dysphagia to solids then liquids. The patient is Arabic, originally born in Egypt, and admitted to a 20 pack-year smoking history, but denied alcohol or illicit drug use. Prior to admission, the patient experienced one episode of hematemesis, in which she vomited specks of frank blood after eating. The patient denied any chest pain, nausea, diarrhea, abdominal pain, and bloody or dark-colored stools. The patient had been on warfarin therapy for the last 15 years and after having blood in her vomitus, her family brought her to the emergency department for further evaluation. The patient’s home medications included ascorbic acid 500 mg daily, calcium carbonate 600 mg daily, losartan 100 mg BID, metoprolol 100 mg BID, simvastatin 40 mg QHS, and warfarin 3 mg daily. Upon presentation, she was admitted to the hospital for further investigation of her upper gastrointestinal bleeding and dysphagia.

**Figure 1:**
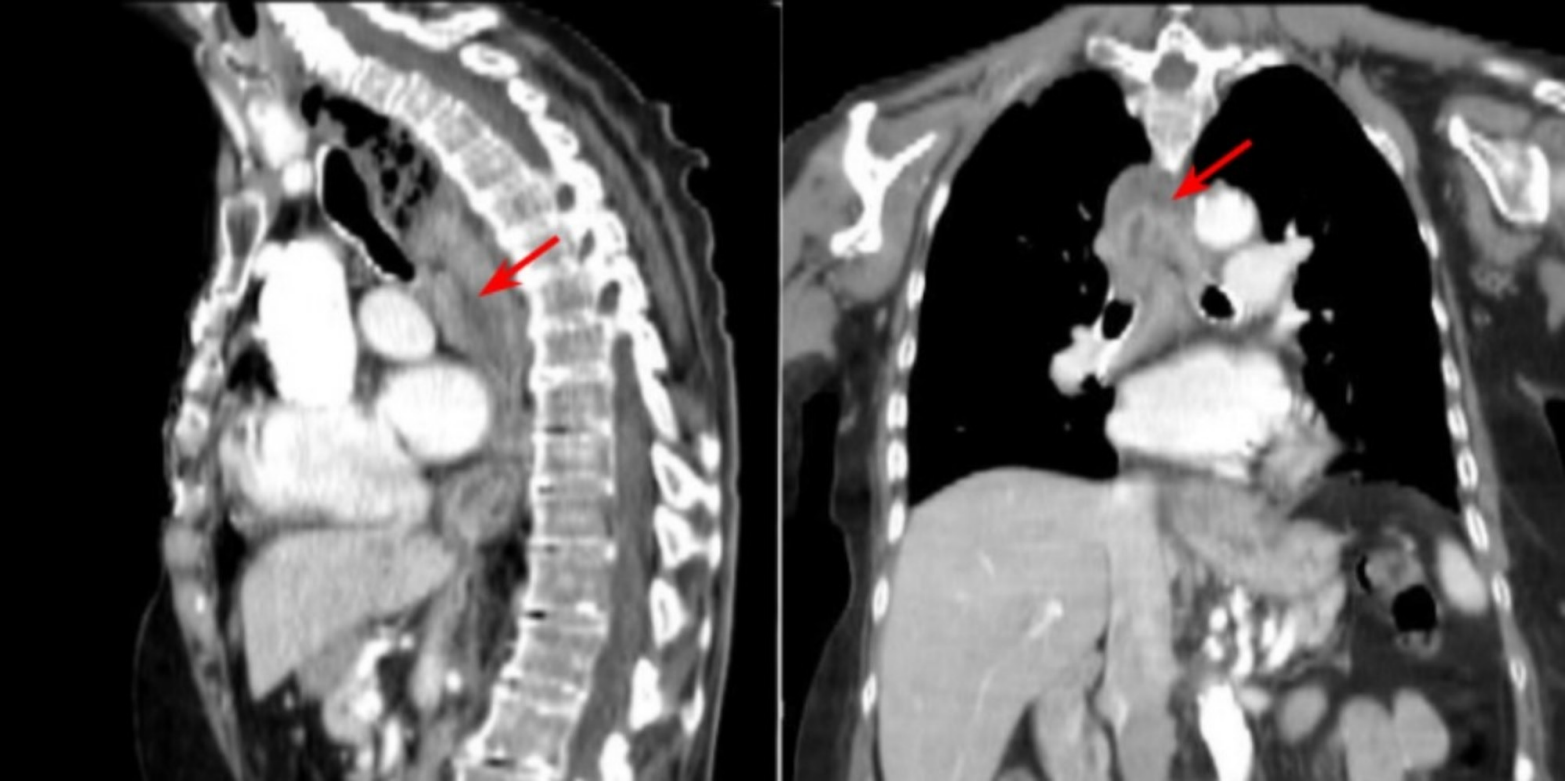
Computed tomography (CT) of the chest with contrast demonstrating marked esophageal dilatation with fluid within the entire length of the esophagus

Initial laboratory studies were significant for normocytic anemia with a hemoglobin of 10.5 g/dL, mean corpuscular volume of 83.3 fL, a prothrombin time of 21.9 seconds and an international normalized ratio of 1.91. Vitals were stable. The patient underwent CT with contrast of the neck and chest which was significant for a large gas and fluid-containing, rounded mass in the posterior mediastinum (Figure 1).

**Figure 2:**
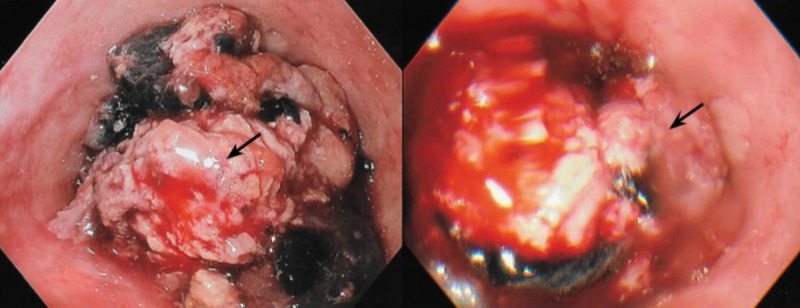
Endoscopic visualization of the obstructing esophageal lesion (black arrow) with thrombus and hemorrhage

Differential diagnosis at this point included a mediastinal mass, Zenker’s diverticulum, esophageal tumor, and achalasia. After abnormal findings were reported on CT imaging, the patient elected to undergo upper endoscopy with possible biopsy of the lesion. Endoscopy was performed and during the procedure, a large, obstructing esophageal mass with adherent clot was visualized in the proximal third of the esophagus (Figure 2).

**Figure 3:**
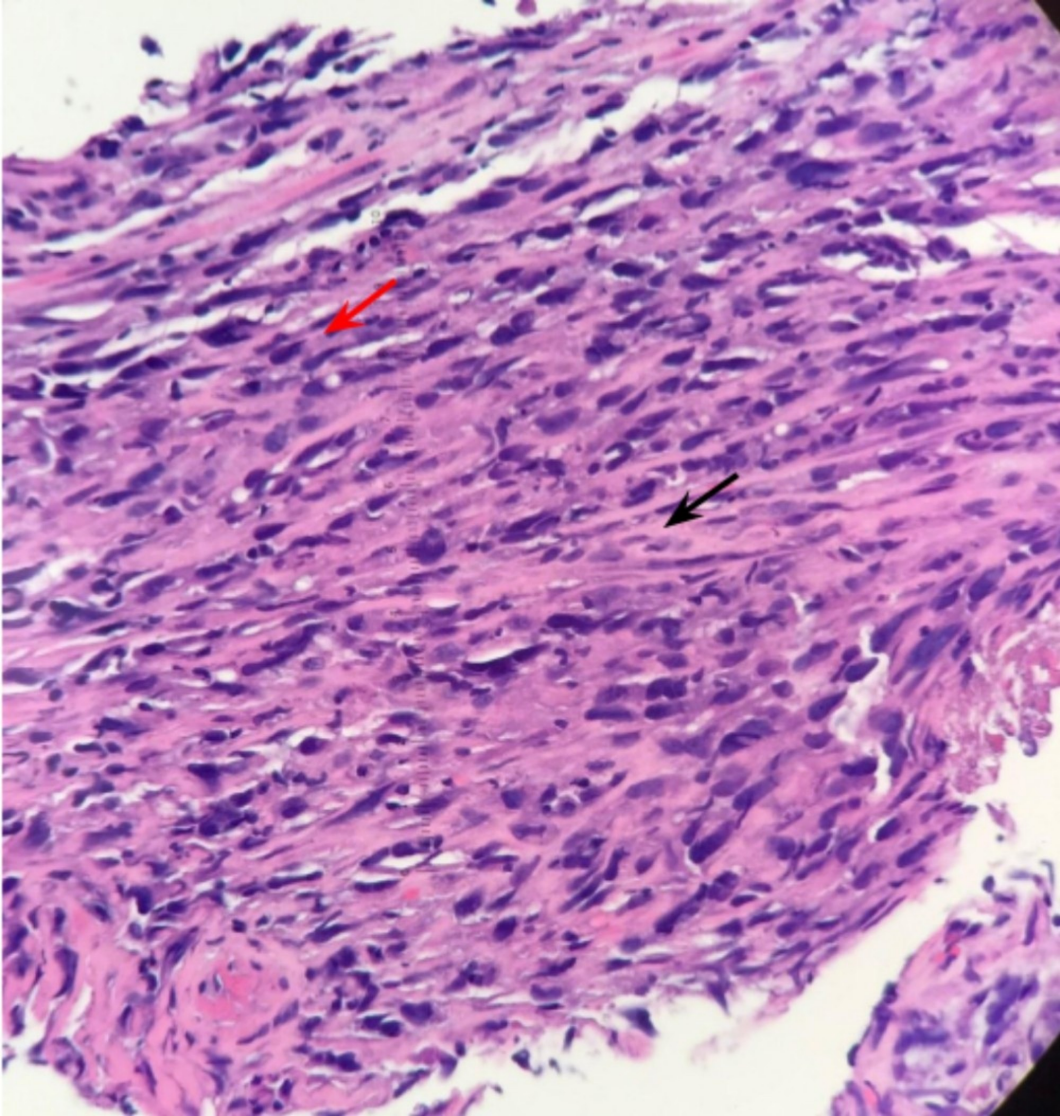
Microscopic evaluation of tumor biopsy demonstrating typical squamous cell carcinoma component (red arrow) and malignant sarcomatoid component (black arrow) as seen in sarcomatoid squamous cell carcinoma

Biopsies of the mass were obtained and the procedure was aborted as the endoscope could not be advanced safely around the mass. Due to the underlying coagulopathy from warfarin therapy and high risk of bleeding of the mass itself, the patient was started on a proton pump inhibitor infusion, which was continued for the duration of her hospitalization. Tissue biopsies from the esophageal mass were fixed and routinely stained (Figure 3).

Pathology results revealed tumor cells demonstrating dual expression with p40 and vimentin immunohistochemical (IHC) stains thus confirming the diagnosis of sarcomatoid squamous cell carcinoma. Smooth muscle actin, CD117, and pan-keratin stains were negative.

After counseling the patient and family about the diagnosis, the patient elected to undergo palliative therapy with the placement of an esophageal stent in order to alleviate her dysphagia. The patient wished not to pursue further diagnostic testing for the purpose of staging the tumor, as she did not want to undergo major surgery, chemotherapy, or radiation therapy for her disease. The patient underwent successful placement of an intraluminal esophageal stent. The patient was monitored in the acute care setting post-operatively and subsequently discharged in stable condition. The patient was instructed to follow up in the outpatient clinic, as she could possibly benefit from radiation therapy in the future.

## Discussion

The majority of esophageal cancers consist of squamous cell carcinomas and adenocarcinomas, as these types account for greater than 90% of all diagnosed cases. [1] In the United States, almost 18,000 new cases of esophageal cancer will be diagnosed each year. [1] Sarcomatoid squamous cell carcinoma of the esophagus is a rare type of esophageal cancer with an incidence from 0.6% to 2% [6]. The histological pattern of sarcomatoid squamous cell carcinoma is characterized by both a carcinomatous and sarcomatous component, including a mixture of malignant spindle cells and squamous cells as seen in Figure 3.

For diagnostic pathology, immunohistochemical biomarkers are used to identify the carcinomatous and sarcomatous components of these tumors [6]. The patient demonstrated pathology indicative of squamous and mesenchymal origin including positive staining for p40, a squamous-specific isoform of the transcription factor p63, and vimentin, a mesenchymal biomarker. 

Due to the rarity of the disorder, large patient studies aimed to evaluate the optimal treatment strategies and overall prognostic factors are lacking. One study by Zhang et al. compared 71 patients with sarcomatoid squamous cell carcinoma of the esophagus to squamous cell to evaluate and classify risk factors, treatment methods, and prognostic indicators. The study demonstrates that sarcomatoid squamous cell carcinoma shows a higher tendency toward locoregional lymphatic spread, indicating a highly malignant potential if not discovered early in the disease [7]. Previously, it was believed that prognosis of sarcomatoid carcinoma is better than that of common squamous cell carcinoma because the sarcomatoid carcinoma tumors tend to grow into the lumen rather than into the wall of the esophagus [3]. However, more recent studies suggest the three-year survival rate of sarcomatoid squamous cell carcinoma is much higher than that of esophageal squamous cell carcinoma but five-year survival is roughly the same [6]. Due to the previous absence of consensus on its histological diagnosis, no standard therapeutic modalities or post-operative surveillance strategies specific to the disease have been determined. 

The subject in this case presentation elected to undergo palliative treatment of her cancer, focused on relieving the dysphagia in order to meet her nutritional needs. Her risk factors included a significant 20 pack-year smoking history, history of breast cancer, and radiation therapy to the chest and mediastinum. The patient denied alcohol use or symptoms of gastritis or reflux disease. Although she may benefit from radiation therapy in the future, no survival benefits have been documented using radiation, chemotherapy, or in combination as these patients typically present in more advanced stages [7]. Genomic testing to identify specific mutant genes for development of target therapies have been published but large studies are lacking [6]. As research in this area progresses, a focus on developing specific therapy regimens and risk factor modification may increase survivability and quality of life for people with this rare form of esophageal cancer.

## Conclusions

Sarcomatoid squamous cell carcinoma of the esophagus is a rare disease that presents challenges in both diagnosis and treatment. Recent research suggests that the disease is of monoclonal origin involving lineages of both mesenchymal and epithelial cell types. Specific risk factors and prognostic indicators have yet to be identified due to the rarity of the disease and lack of large cohorts for study. Treatment modalities are selected based on the extensiveness of disease and comorbidities of the patient, although none have been shown to significantly improve survivability in advanced disease.
